# High-purity recycling of hematite and Zn/Cu mixture from waste smelting slag

**DOI:** 10.1038/s41598-020-66077-8

**Published:** 2020-06-03

**Authors:** Yang Huo, Xiang Song, Suiyi Zhu, Yu Chen, Xue Lin, Yaqiong Wu, Zhan Qu, Ting Su, Xinfeng Xie

**Affiliations:** 1 Science and Technology Innovation Center for Municipal Wastewater Treatment and Water Quality Protection, Northeast Normal University, Changchun, 130117 China; 2 Jilin Institute of Forestry Survey and Design, Changchun, 130022 China; 3 School of Forest Resources and Environmental Science, Michigan Technological University, Houghton, MI 49932 United States

**Keywords:** Environmental monitoring, Environmental impact, Environmental chemistry

## Abstract

In this study, Zn/Cu-bearing smelting slag was recycled via an integrated acid dissolution and hematite precipitation method. The slag was dissolved in nitric acid to generate an acid solution containing 23.5 g/L Fe, 4.45 g/L Zn and 2.81 g/L Cu, which was subjected to hydrothermal treatment with the addition of levulinic acid (LA). More than 99.95% of the initial Fe content was removed as hematite particles with diameters of approximately 200 nm, and the residual Fe concentration in the acid was 0.43 mg/L. The generated hematite contained 97.3% Fe_2_O_3_, 0.64% ZnO and 0.58% CuO. Greater than 99% of the initial Zn and Cu was retained in the acid and further precipitated as Zn/Cu-bearing solids by adjusting the solution pH to 9. The precipitated Zn/Cu-bearing solids contained 33.6% Zn and 21.7% Cu, whereas the Fe content was less than 0.2%. This paper is the first report of an environmentally friendly approach for recycling smelting slag without generating any hazardous waste.

## Introduction

Smelting slag is a typical hazardous waste generated in the smelting and metallurgy industry^1,2^. Slag contains high concentrations of heavy metals, such as Cu, Zn, Cr and Ni, which are harmful if released into the environment^3^. In China, smelting slag is disposed of as an environmental priority pollutant, wherein the slag is generally stabilized with cement before landfilling for safety purposes^4^. Although smelting slag is a potential source of various alloys^5–7^, it is difficult to recycle slag due to its complex impurities, such as Fe, Al, Si, Ca and high-molecular-weight organic matter, which are mainly derived from flocculants in smelting wastewater treatment^7,8^. The conventional method for recycling heavy metals from smelting slag is chemical leaching with a strong acid^5–7,9^. Most heavy metals in the slag are dissolved by strong acid leaching, followed by the dissolution of impurities (e.g., Fe and Al). However, this method has two main disadvantages. First, previous studies have shown that Fe/Al impurities are easily complexed with extraction agents and/or coprecipitated with heavy metals in the recycling process, thereby decreasing the purity of the recoverable heavy metals^10,11^. Second, it has been reported that Si/Ca compounds and high-molecular-weight organic matter in slag do not completely dissolve; instead, these materials are commonly complexed with residual heavy metals to generate hazardous undissolved solid waste^12^, which should be properly treated before landfilling. Thus, the strong acid leaching method needs to be modified to effectively recycle heavy metals without generating hazardous waste. Studies have revealed that the impurities in slag are mainly introduced by the addition of flocculants, such as polyaluminum chloride, polyferric sulfate, polysilicic acid and polyacrylamide, during smelting wastewater treatment^13–15^. Therefore, we hypothesized that in smelting wastewater treatment, the use of a single flocculant might effectively reduce impurities in the leaching acid and eliminate the undissolved solid.

In leaching acid, Fe is in the ferric form and can be removed through chemical precipitation^16^ and/or hydrothermal methods^6,7,17^, resulting in a high concentration of heavy metals in the acid. For example, nearly 100% of Fe was removed as Fe oxyhydroxide when the acid was adjusted to a pH of 4^16,18^. However, the formed Fe oxyhydroxide had a sufficient number of hydroxyl groups for heavy metal coordination, resulting in a low retention rate of heavy metals in the acid^9^. Compared with chemical precipitation, the hydrothermal method exhibits a higher retention rate of heavy metals. Lu *et al*. found that after Fe/Cu-bearing hydrochloric acid was hydrothermally treated at 155 °C for 60 min, less than 0.1% of the Cu was removed, whereas nearly 30% of total Fe was removed as hematite^7^. With the addition of H_2_O_2_, the total Fe removal rate further increased to 90.7%^6^. Despite the effective removal of Fe from the leaching acid, the residual Fe was highly concentrated (nearly 1.5 g/L); hence, further separation was required before heavy metal recycling^17^.

The objective of this study was to develop a novel route to recycle heavy metals (e.g., Zn and Cu) from smelting slag without generating any secondary waste. The study also aimed to optimize the recycling conditions to obtain high-purity Zn/Cu products.

## Materials and methods

### Smelting slag treatment

Smelting wastewater was discharged from the electroplating workshop of Sanhe Company (Changchun, Jilin, China), in which the residual Zn and Cu concentrations were 30.7 and 19.3 mg/L, respectively. The smelting wastewater was treated by coagulation through the addition of commercial FeCl_3_ 6H_2_O. Crystallized FeCl_3_ 6H_2_O was added to approximately 2 t of wastewater, which was stored in a flocculation tank, at a dosage of approximately 0.8 g/L. Subsequently, the wastewater was adjusted to a pH of 7.5 through the addition of caustic soda under constant stirring at 70–90 rpm for 1 h followed by 24 h of precipitation. A yellowish precipitate was generated at the bottom of the tank, which was pumped to a plate and frame filter press (XAMY6/450-30U, Runnan-Shanghai, China) to mechanically dewater. Thus, brownish sludge cake was generated.

Samples were taken from the smelting slag, and then the sampled slag was completely dissolved in nitric acid solution at room temperature for one week. The leaching solution contained 23.5 g/L Fe, 4.45 g/L Zn, 2.81 g/L Cu, 61.8 g/L NO_3_^-^ and 3.75 g/L Cl^-^.

### Fe separation experiments

Highly purified Fe oxides were successfully separated from the leaching solution via a one-step hydrothermal route. First, 20 mL of leaching solution was adjusted to a pH of 0.4 through the addition of 5 M NaOH solution, after which the solution was dumped into a 50 mL Teflon vessel. The amount of Fe in the vessel was approximately 8.39 mmol. Second, 16.79 mmol levulinic acid (LA) was directly added to the vessel, thereby achieving a M_LA_/M_Fe_ ratio of 2. Third, the vessel was sealed, placed in a drying oven (DHG-9030A, Yiheng, Shanghai, China), heated to 220 °C for 6 h and then water-cooled to room temperature. Fourth, the supernatant and deposit were collected separately and then freeze-dried overnight at −80 °C. Control experiments were also performed by decreasing the M_LA_/M_Fe_ ratio from 2 to 1, 0.5, 0.25 and 0. The concentrations of Fe, Zn and Cu in the supernatant were measured by inductively coupled plasma optical emission spectrometry (ICP-OES, Avio-200, Perkinelmer, USA). To optimize the hydrothermal treatment time for Fe precipitation, time-course experiments were also carried out according to the abovementioned procedures. The hydrothermal treatment time was reduced from 6 to 4, 2, 1 and 0.5 h, and the corresponding deposit and supernatant were also characterized.

At an M_LA_/M_Fe_ ratio of 1, nearly 100% Fe removal was achieved. Thus, the residual Zn and Cu in the supernatant was recycled by directly adjusting the supernatant to a pH of 9 through the addition of 5 M NaOH. The generated deposit was collected and then washed three times with deionized water, followed by drying at 105 °C for 5 h. The Fe, Zn and Cu concentrations in the residual supernatant were also determined by ICP-OES.

All experiments in this study were performed in triplicate, and the average of the values was reported. Various sets of the experimental data were subjected to one-way analysis of variance using Microsoft Excel (v. 2016, Microsoft, USA), and the differences between the sets of data were not statistically significant.

### Characterization

The deposited particles were characterized using scanning electron microscopy (SEM, JSM-6400, Jeol, Japan), X-ray diffraction (XRD, Rigaku, Rint2200, Japan) and X-ray fluorescence (XRF, S4-Explorer, Bruker, Germany). The nitrate and nitrite in the supernatant were determined by ion chromatography (881 Pro, Metrohm, Switzerland). The pH value and total organic carbon (TOC) were measured using a pH meter (S210-S, Mettler Toledo, USA) and a TOC analyzer (TOC 500, Shimadzu, Japan), respectively.

## Results and discussion

### Optimization of LA dosage

**Figure 1:**
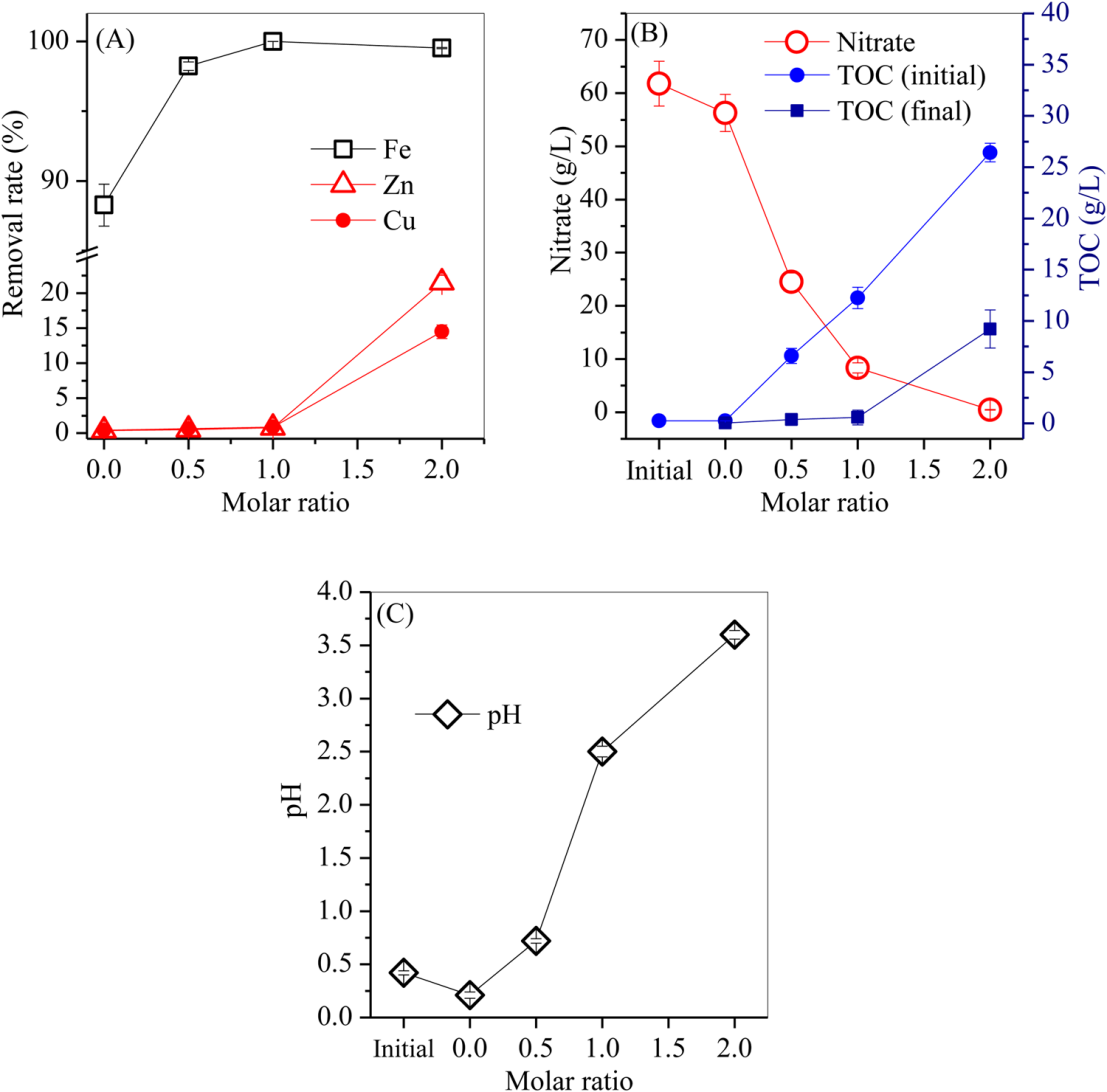
Variations in the (**A**) removal rates of Fe, Zn and Cu, the (**B**) concentrations of nitrate and TOC, and the (**C**) pH values with increasing LA dosage.

Fe was efficiently separated from the leaching acid, as shown in Fig. 1. Without LA, the Fe removal rate reached 88.3%, whereas the removal rates of Zn and Cu were both less than 0.5% (Fig. 1(A)). Additionally, the nitrate concentration decreased from 61.8 g/L to 56.3 g/L (Fig. 1(B)), and the solution pH slightly decreased from 0.4 to 0.2. By adding LA, the total Fe removal rate significantly increased to 98.2% at a molar ratio (M_LA_/M_Fe_) of 0.5, peaked at nearly 100% at a molar ratio of 1, and slightly decreased to 99.5% at a molar ratio of 2. Accordingly, the Zn and Cu removal rates slightly increased from 0.52% and 0.66% at a molar ratio of 0.5 to 0.78% and 0.84% at the molar ratio of 1, whereas they substantially increased to 21.5% and 14.5% at a molar ratio of 2. Therefore, the optimal molar ratio was 1 for highly efficient separation of Fe from leaching acid, in which the Fe removal rate was nearly 100% (the residual Fe concentration was 0.43 mg/L) and the Zn and Cu removal rates were both less than 1%.

The decreases in the TOC and nitrate content were also observed. For instance, at a molar ratio of 1, the concentration of the residual nitrate was 8.3 g/L, whereas the concentration of the residual TOC was 0.6 g/L, suggesting that the added LA was inadequate to completely oxidize nitrate. When the molar ratio increased from 1 to 2, the nitrate concentration decreased to 0.47 g/L, whereas the residual TOC concentration increased to 9.2 g/L, demonstrating that approximately 99.2% of the nitrate was removed by the excessive dosage of LA. As the LA dosage increased, the solution pH increased to 2.5 at a molar ratio of 1 and further increased to 3.6 at a molar ratio of 2, indicating that H^+^ was involved in the redox reaction between LA and nitrate. The variation of TOC and nitrate were subjected to the reaction between LA and nitrate under hydrothermal conditions (these conditions will be discussed in Section 3.4).

**Figure 2:**
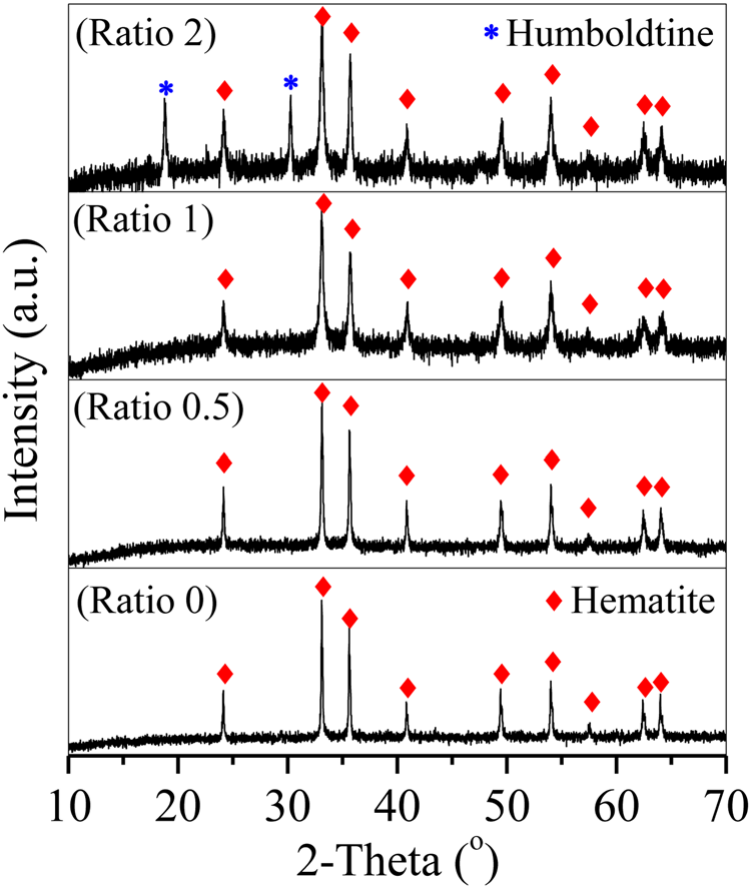
XRD patterns of the precipitated hematite particles generated at molar ratios ranging from 0 to 2.

**Figure 3:**
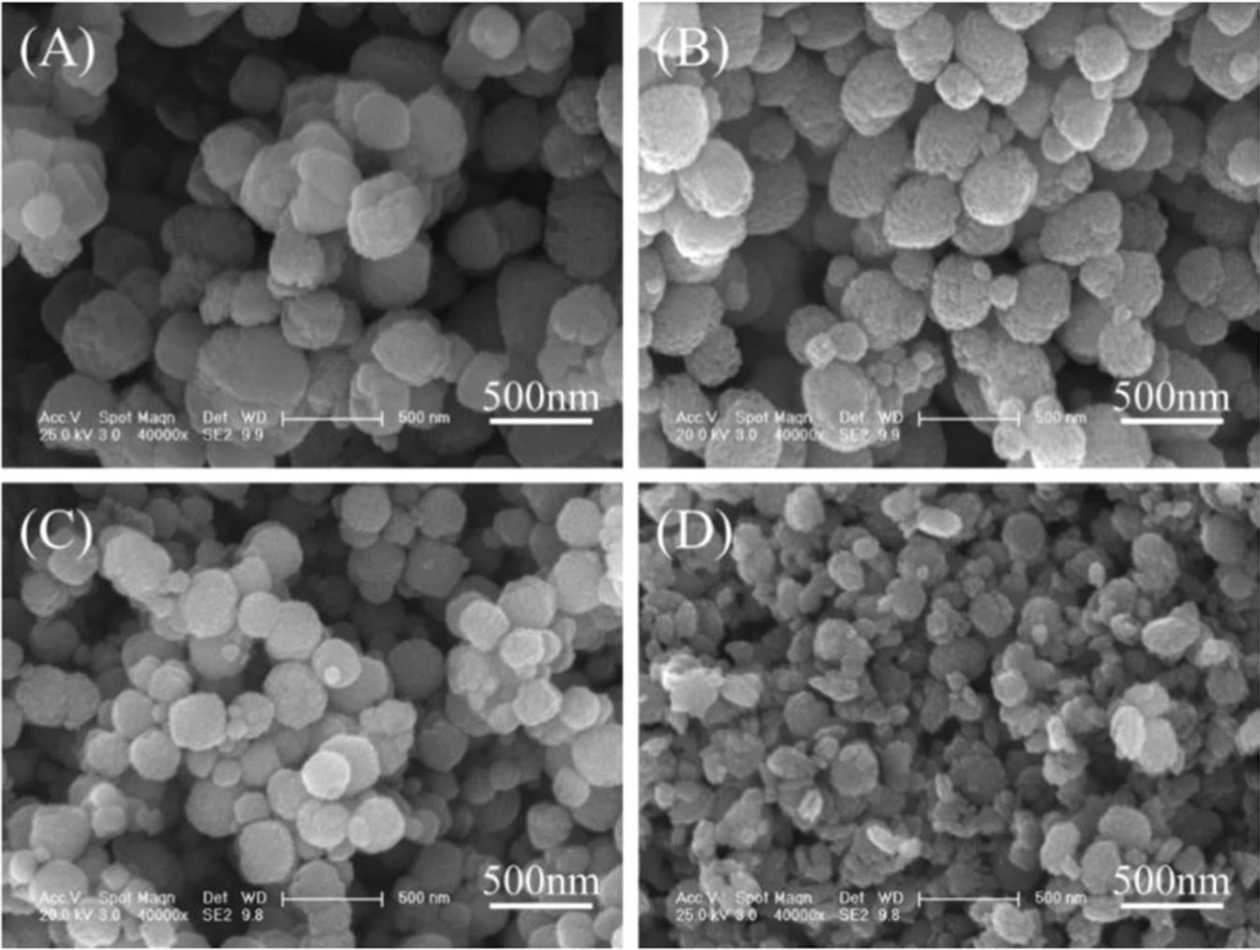
SEM images of the precipitated particles generated at molar ratios of (**A**) 0, (**B**) 0.5, (**C**) 1 and (**D**) 2.

Without the addition of LA, Fe was removed as hematite particles (Fig. 2, molar ratio = 0) with diameters of 300–500 nm (Fig. 3(A)). After adding LA, sharp peaks of hematite were also observed in the curves of the particles generated at molar ratios of 0.5 and 1 (Fig. 2); however, the diameter of the particles gradually decreased to approximately 200 nm as the molar ratio increased to 1 (Fig. 3(B,C)). A previous study revealed that LA and its oxidized intermediates were electrostatically adsorbed on the positively charged surface of hematite particles during hydrothermal treatment, which inhibited the aggregation and crystal growth of hematite particles and resulted in smaller hematite particles than those observed without the addition of LA^19^. When the molar ratio was 2, the obtained particles showed sharp peaks of hematite and new peaks at 2θ = 18.8° and 30.3° corresponding to humboldtine (Fig. 2, molar ratio = 2); hence, the obtained particles were a mixture of spherical hematite particles and small humboldtine chips (Fig. 3(D)).

**Figure 4:**
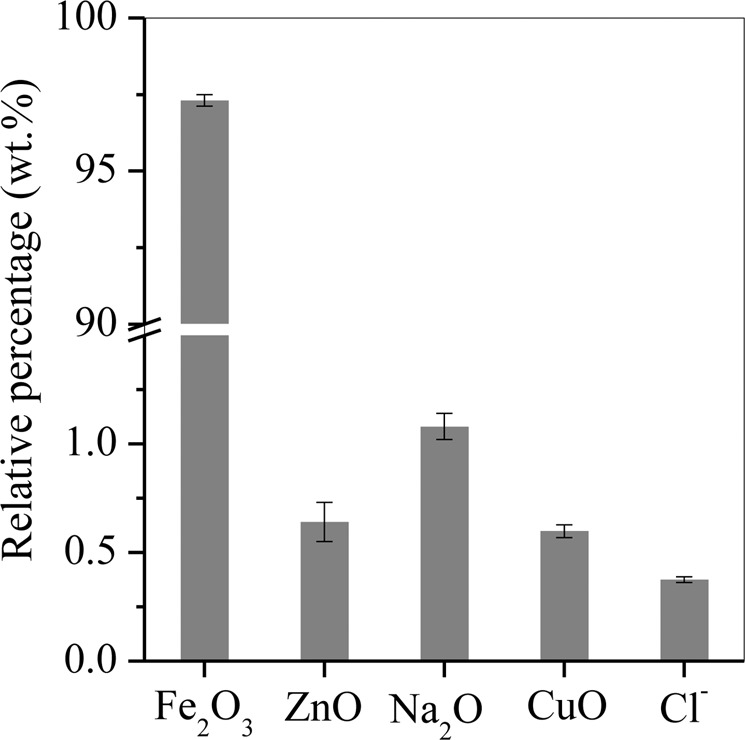
Major composition of the precipitated hematite particles generated with the optimal LA dosage and 6 h of hydrothermal treatment.

To analyze the components of the generated hematite with the optimal LA dosage, an XRF experiment was performed (Fig. 4). The precipitated hematite contained 97.3% Fe_2_O_3_, 0.64% ZnO and 0.58% CuO, which is highly purified and can subsequently be used as a raw material in the dye and electronic industries.

### Optimization of the hydrothermal treatment time

**Figure 5:**
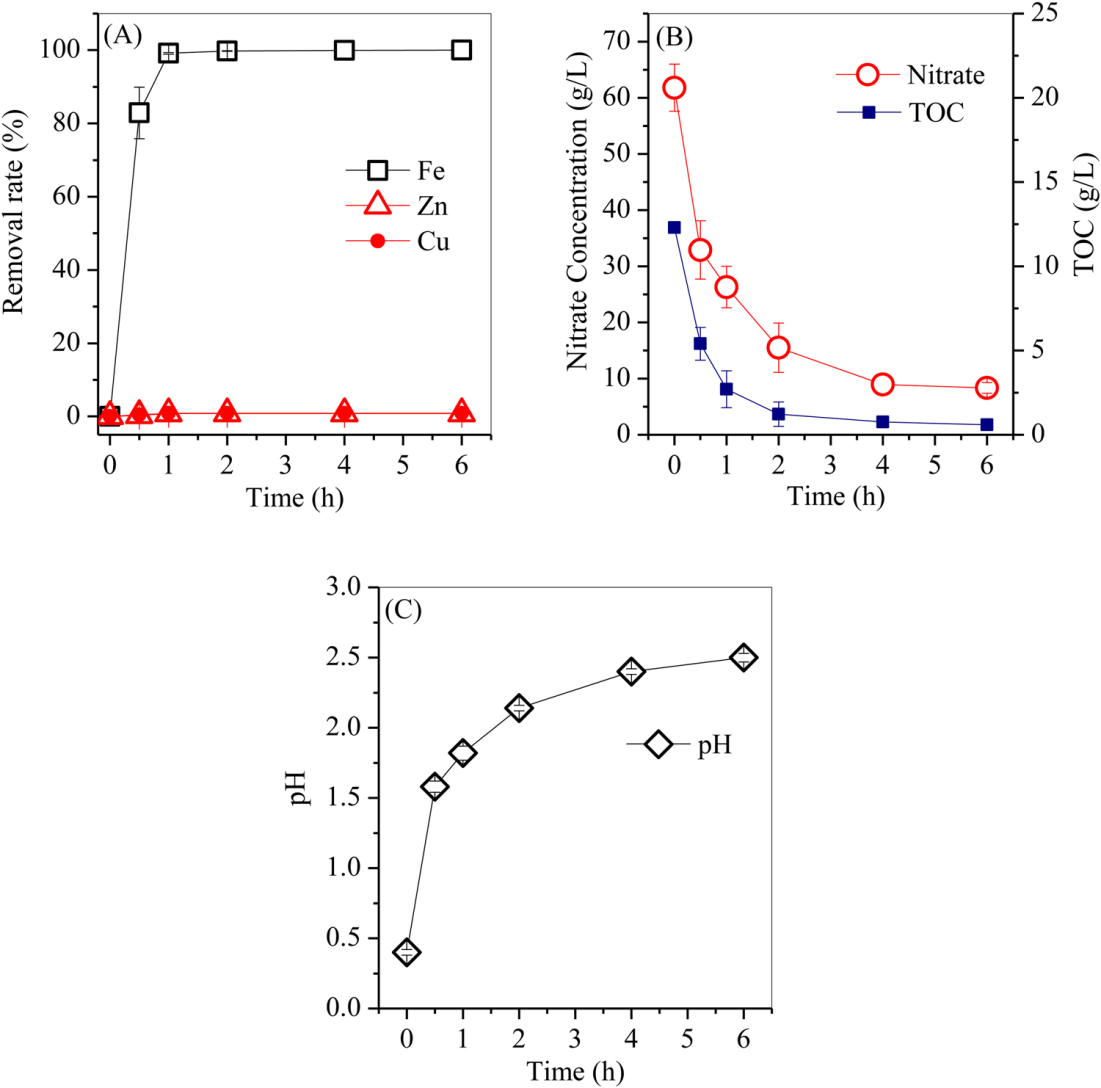
Variations in the (**A**) removal rate of Fe, Zn and Cu, (**B**) concentrations of nitrate and TOC and (C) pH as the hydrothermal treatment time extends from 0 to 6 h.

The time course of Fe removal from the leaching acid with different hydrothermal treatment times was investigated, as shown in Fig. 5. The Fe removal rate increased to 82.3% after 0.5 h of treatment, then further increased to 99.2% after 1 h of treatment, and became steady at nearly 100% as the treatment time extended to 6 h. The Zn and Cu removal rates were 0.31% and 0.43% after 0.5 h of treatment, followed by slight increases to 0.78% and 0.84% after 1 h of treatment, and then remained nearly constant as the treatment time extended to 6 h. These results revealed that the hydrothermal treatment time is an important factor for Fe precipitation. With extension of the hydrothermal treatment time, the nitrate and TOC concentrations gradually decreased from 61.8 g/L and 12.3 g/L after 1 h of treatment to 8.33 g/L and 0.95 g/L after 6 h of treatment on account of the redox reaction between the nitrate and TOC. Consequently, the solution pH gradually increased from the initial 0.4 to 1.5 after 0.5 h of treatment and finally to 2.5 after 6 h of treatment.

**Figure 6:**
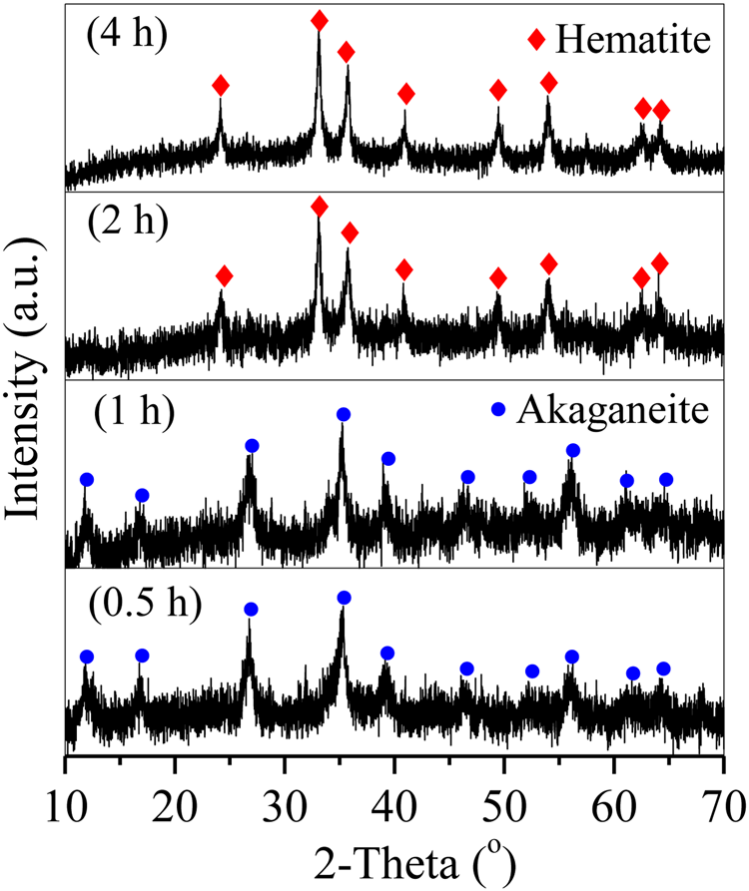
XRD patterns of the particles generated at hydrothermal treatment times of 0.5 h, 1 h, 2 h, and 4 h.

**Figure 7:**
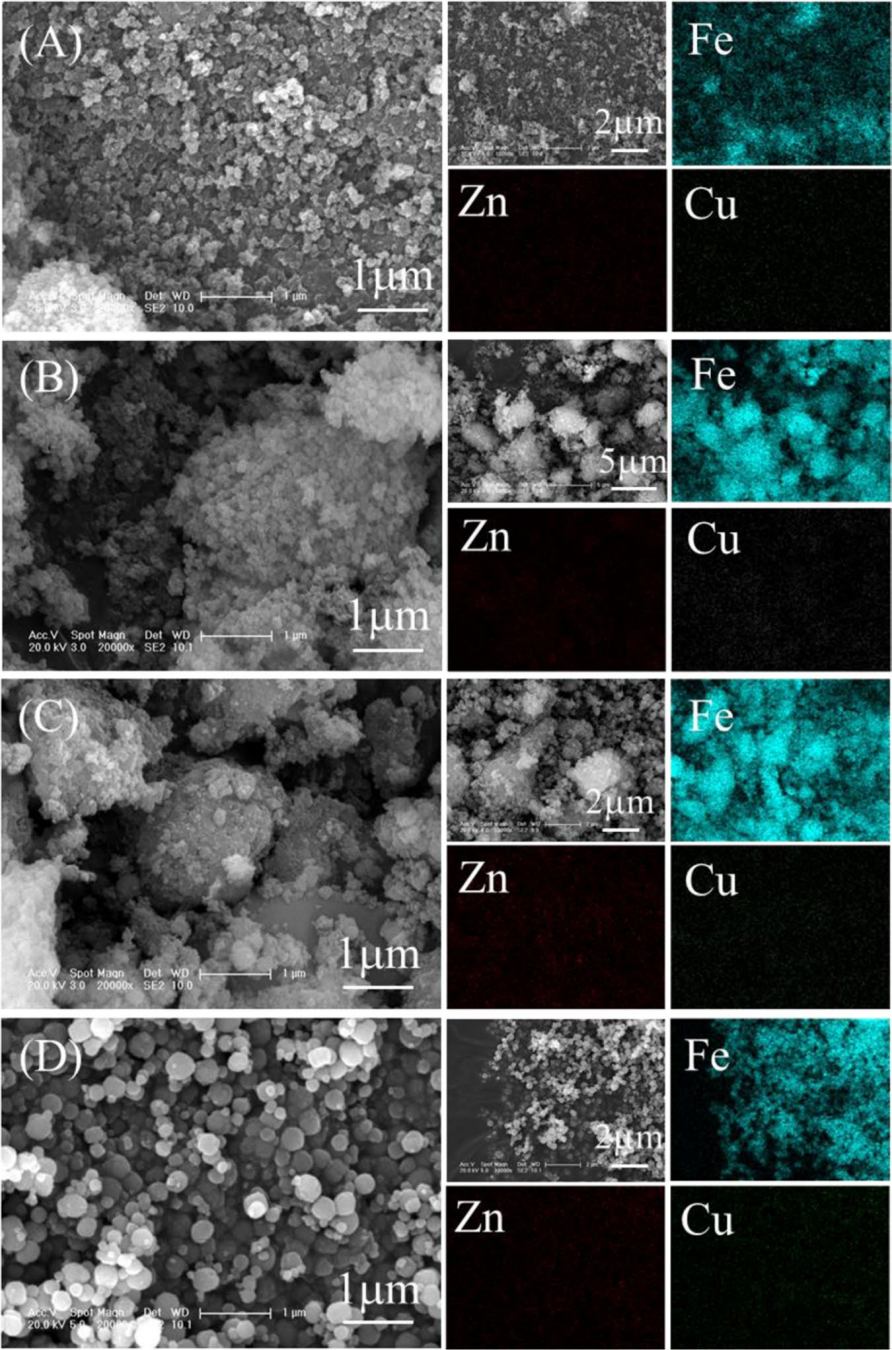
SEM images of the hematite particles generated at hydrothermal treatment times of (**A**) 0.5 h, (**B**) 1 h, (**C**) 2 h, and (**D**) 4 h.

With hydrothermal treatment, Fe precipitated into irregular akaganeite aggregates with uniform distributions of Fe and blurred distributions of Zn and Cu during the first 1 h of treatment [Fig. 6 (0.5 h and 1 h) and Fig. 7(A,B)], subsequently transformed into hematite aggregates after 2 h of treatment [Fig. 6 (2 h) and Fig. 7(C)], and finally formed into spherical hematite particles with diameters of approximately 200 nm after 4 h of treatment. These results demonstrated the precipitation of Fe as hematite spheres with akaganeite aggregates as the intermediate. The formation of akaganeite will be discussed in Section 3.4.

### Recycling of Cu/Zn-bearing particles

**Figure 8:**
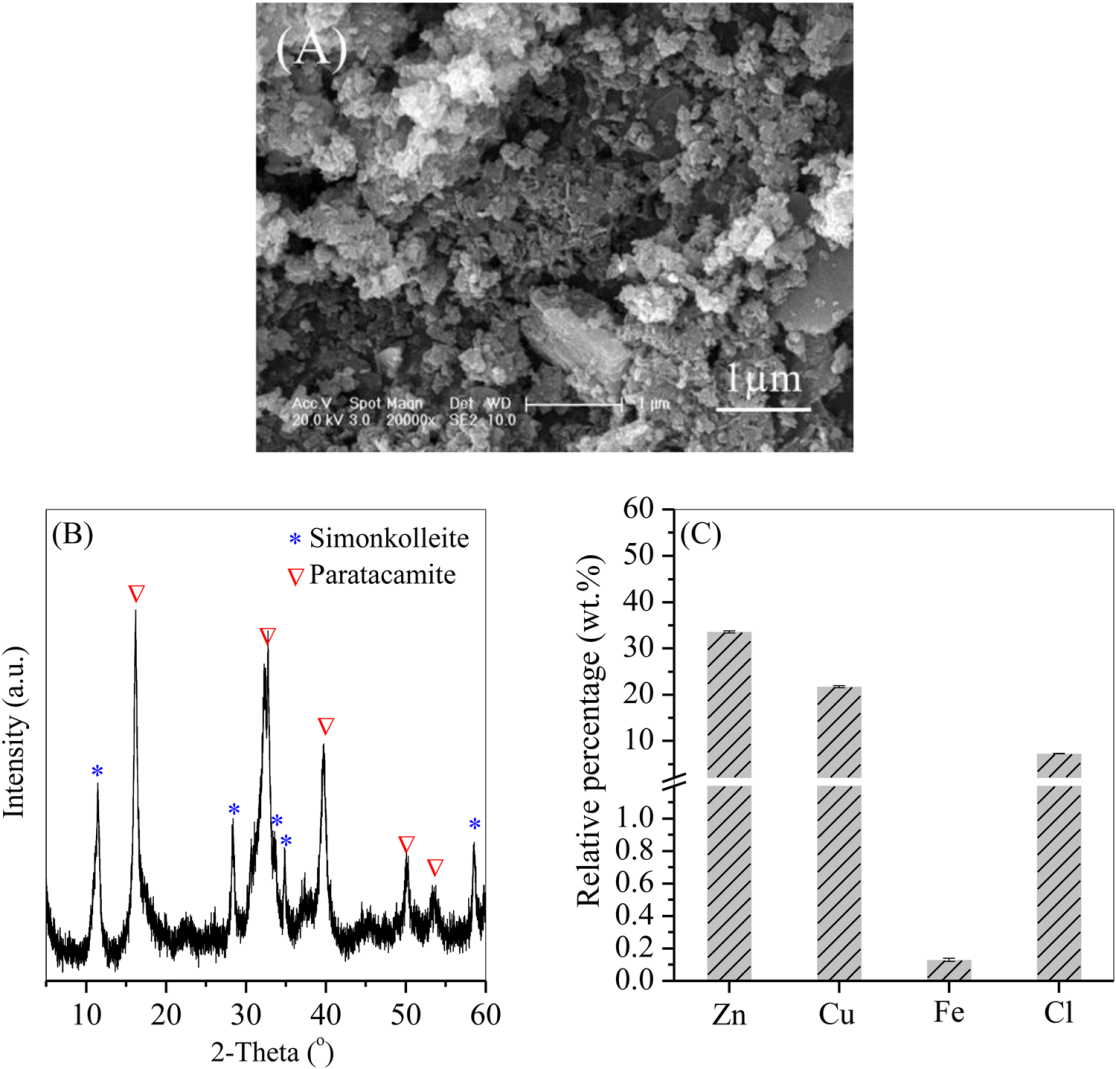
(**A**) SEM image, (**B**) XRD pattern and (**C**) major composition of the precipitated Cu/Zn mixture.

After Fe was removed from the leaching solution, the residual Zn and Cu concentrations were 4.42 and 2.78 g/L, respectively; these values are approximately 140 times those in the smelting wastewater. The residual Cu and Zn were recovered as the Cu/Zn-bearing irregular aggregates (Fig. 8(A)) by adjusting the solution pH to 9. The Cu and Zn precipitated as a mixture of simonkolleite and paratacamite (Fig. 8(B)), in which the relative weight percentages of Zn and Cu were 33.6% and 21.7%, respectively, whereas that of Fe was less than 0.2%. These results demonstrated that the Cu/Zn mixture was highly purified.

### Separation mechanism of Fe from the leaching acid

Fe was abundant in the leaching solution and was spontaneously hydrolyzed in three steps under hydrothermal conditions. First, Fe was hydrolyzed to weakly crystallized Fe oxyhydroxides (Eq. (1)), such as Fe^2+^OH, Fe^+^(OH)_2_ and Fe(OH)_3_^20,21^. Second, the conjunction of two adjacent hydroxyl groups on each Fe oxyhydroxide occurred, to release one water molecule, with the formation of an Fe-O-Fe bond^22,23^. Third, as the above reaction continued, akageneite was generated and further transformed into hematite (Eq. (2)).

1 $$F{e}^{3+}+3{H}_{2}O\to Fe{(OH)}_{3}+3{H}^{+},$$

2 $$2Fe{(OH)}_{3}\to F{e}_{2}{O}_{3}+3{H}_{2}O,$$

**Figure 9:**
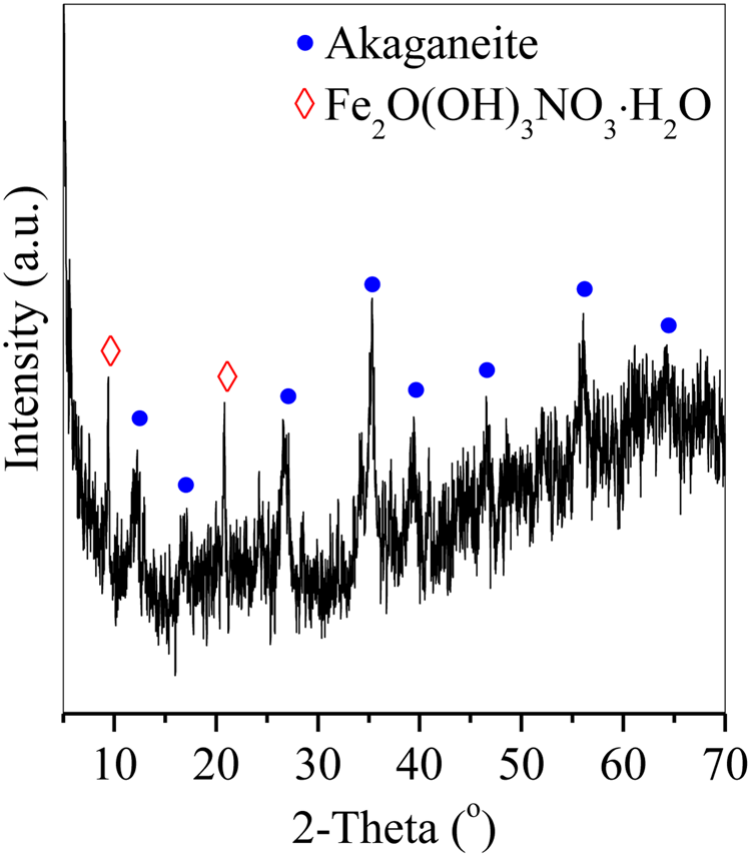
XRD pattern of the hematite particles generated after 0.2 h of hydrothermal treatment.

To further analyze the hydrolysis of Fe, the leaching solution was hydrothermally treated without LA according to the method in Section 2.2, and then the particles generated after 0.2 h of treatment were characterized by XRD (Fig. 9). The typical peaks of Fe_2_O(OH)_3_NO_3_ H_2_O and akaganeite were observed in the curve of the hydrolyzed product of Fe after 0.15 h of hydrothermal treatment, demonstrating that the Fe-O-Fe bond was generated from the polymerization of the initial hydrolyzed product (e.g., Fe_2_O(OH)_3_NO_3_ H_2_O) with akageneite as the intermediate. Elmasry *et al*. investigated the thermal decomposition of Fe(NO_3_)_3_ 9H_2_O and found that the major intermediates were Fe(OH)(NO_3_)_2_ and Fe(OH)_2_NO_3_^21^ with hematite as the final product, which is similar to the findings in this study.

During Fe hydrolysis, OH^-^ was involved in the formation of Fe oxyhydroxides. The residual H^+^ led to a decrease in solution pH from 0.4 to 0.2 (Fig. 1(C)). In the leaching acid, the coprecipitation of Fe and Zn/Cu did not occur. When the free Zn and Cu were coordinated on the surface hydroxyl group (≡Fe-O-H) of the Fe oxyhydroxides, the polymerization of two adjacent Fe oxyhydroxides was inhibited. However, H^+^ was rich in the leaching acid and easily replaced the coordinated Zn/Cu on the Fe oxyhydroxides, which regenerated the hydroxyl group, thereby promoting the polymerization reaction of Fe oxyhydroxides with hematite as the final product. During the hydrothermal treatment process, H^+^ accumulated in the acid and finally remained constant, in which the hydrolysis of Fe was in equilibrium, which led to a relatively high level of residual Fe. In addition, nitrate was also hydrothermally decomposed to NO_2_ and O_2_^24^ via Eq. (3), which caused a decrease in nitrate concentration (Fig. 1(B)).

3 $$2N{O}_{3}^{-}\to 2N{O}_{2}+{O}_{2},$$

**Figure 10:**
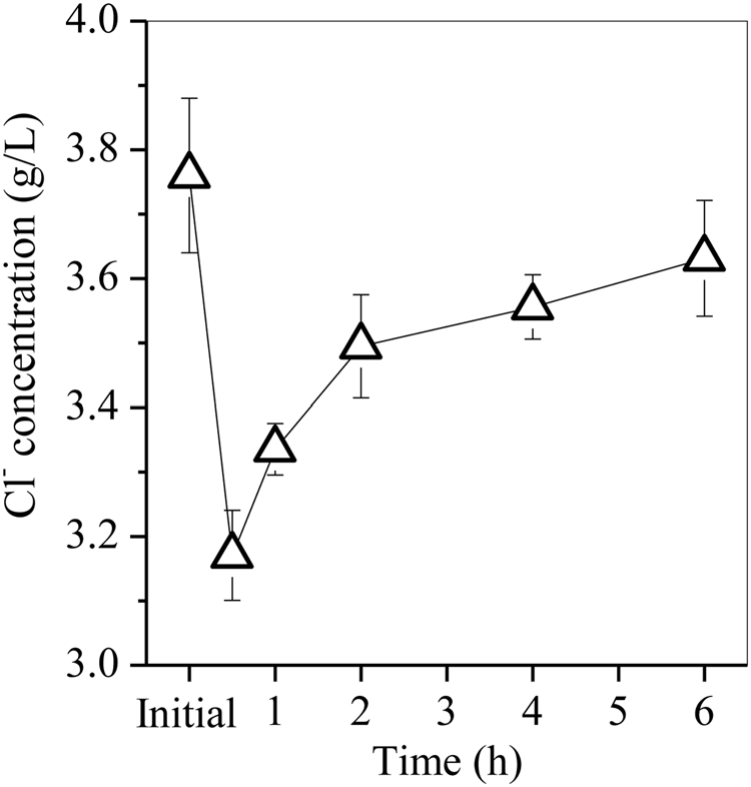
Concentration of Cl^-^ in the supernatant after hydrothermal treatment.

When LA was introduced in the leaching acid, the redox reaction between LA and nitrate occurred via Eq. (4). The carboxyl group on the side chain of the LA molecule was prone to losing electrons via a decarboxylation reaction with the generation of 2-butanone and methyl vinyl ketone^25^, which was further oxidized to oxalic acid with CO_2_ and H_2_O as the final product. After hydrothermal reaction with an M_LA_/M_Fe_ ratio of 2, the supernatant was collected, and then the residual concentrations of 2-butanone and methyl vinyl ketone in the supernatant were determined by headspace-gas chromatography (HS-GC, QP2010-Ultra, Shimadzu, Japan) following the method of Gong *et al*.^26^, whereas the oxalic acid concentration was detected by high-performance liquid chromatography (HPLC, LA-20, Shimadzu, Japan) in accordance with Pelin *et al*.^27^. The concentrations of 2-butanone and methyl vinyl ketone were 23.9 and 0.07 mg/L, respectively, which were both lower than that of oxalic acid (282.4 mg/L). This demonstrated the generation of 2-butanone, methyl vinyl ketone and oxalic acid in the reaction between LA and nitrate. Similar intermediates of 2-butanone and methyl vinyl ketone were also observed in the oxidative decarboxylation of LA by Ag^+^/S_2_O_8_^2-^ after autoclaving at 100 °C for 0.5 h^26^. As the LA continued to be consumed, both TOC and nitrate concentrations decreased, whereas the solution pH increased, which promoted the further hydrolysis of Fe to Fe oxyhydroxides. The newly generated Fe oxyhydroxides were steadily transformed to akageneite in the presence of a sufficient amount of Cl^-^. Figure 10 shows that the initial concentration of Cl^-^ was 3.75 g/L, which decreased to 3.17 g/L after 0.5 h of hydrothermal treatment and then gradually increased to 3.63 g/L as the hydrothermal treatment time extended to 6 h. These findings suggest that Cl^-^ was involved in the formation of akageneite.

4 $${C}_{5}{H}_{8}{O}_{3}+4N{O}_{3}^{-}+2{H}^{+}\to 2{N}_{2}+5C{O}_{2}+5{H}_{2}O,$$

Akageneite has a tetragonal structure, in which the inner tunnels parallel to the c-axis of the tetragonal lattice are partially occupied by Cl^-^, F^-^ and OH^-^^28^ for structural crystal stability^29,30^. When the hydrothermal treatment time was longer than 2 h, the *in situ* conversion of akaganeite to hematite occurred with the release of water molecules and Cl^-^ in the tunnels^31,32^, which was in agreement with the release of Cl^-^ in the conversion of akageneite to hematite (Fig. 10). Therefore, the Fe precipitated as Fe oxyhydroxides and finally converted to hematite with akageneite as the intermediate.

### Potential application

For safety purposes, smelting slag is commonly collected by a professional waste company and stabilized with cement before landfilling. According to the price provided by Lantian Waste Ltd. (Changchun, China), the total cost for disposing 1 ton of smelting slag is approximately US$ 1304.3, which could be saved by recycling Cu/Zn-bearing products. The reuse of 1 ton of smelting slag requires power (US$ 92.4) and approximately 1.68 tons of nitric acid, 0.44 tons of LA, and 0.486 tons of caustic soda, bringing the total cost to US$ 1496.7. However, costly LA can be replaced by a cheap organic acid, and the product of the hematite and Zn/Cu mixture is highly marketable, which can reduce the cost of slag reutilization. Therefore, the processing method for recycling Zn/Cu from smelting slag has potential applications.

The method described in this study has two advantages in the disposal of smelting slag. One advantage is the effective removal of Fe from the leaching acid, which produces hematite nanoparticles with a purity of 97.3%. The other advantage is the low residual Fe content and high retention of Zn and Cu in the leaching acid. Thus, the highly purified Zn/Cu product was recycled without generating any secondary waste. The recycled Zn/Cu precipitates contained 33.6% Zn and 21.7% Cu with less than 0.2% Fe. The composition meets the guidelines for the active pharmaceutical ingredients of commercial Zn/Cu oxyhydroxide^33,34^, demonstrating the economic value of the recycled Zn/Cu mixture. In addition, the Zn/Cu precipitates can be easily divided into two purified solutions (Zn-bearing or Cu-bearing) via purification methods, including liquid membrane^35^, resin extraction^36^, or solvent extraction^37^, and then directly reused as raw materials in the electroplating process. Other heavy metals, such as Ni, Pb and Cd, showed similar chemical performance to Zn and Cu. These other metals can also be precipitated from wastewater as slag through the addition of a polyferric chloride flocculant, which can be recycled to produce highly purified heavy metal-bearing products. Furthermore, the removal of Fe impurities in this study may shed light on promoting the extraction efficiency of valuable materials (e.g., rare earth metals) from metal-bearing solutions and wastewater. Future studies should focus on the separation of Fe with the addition of low-cost organic matter and the effectiveness of recycling other heavy metals.

## Conclusion

Zn/Cu-bearing smelting wastewater was treated by chemical coagulation with polyferric chloride to generate Zn/Cu smelting slag. The slag was dissolved in nitric acid to generate an Fe/Zn/Cu-bearing solution. Through an optimized hydrothermal treatment (6 h), Fe was precipitated as hematite nanoparticles with a purity of 97.3%, and the residual Fe concentration in the acid was 0.43 mg/L; the removal rate was less than 1% for both Zn and Cu. The concentrations of Zn and Cu in the acid were 4.42 and 2.78 g/L, respectively; these concentrations are approximately 140 times those in the smelting wastewater. Zn/Cu in the acid was further precipitated by adjusting the solution pH to 9, and the final relative weight percentages of Zn and Cu were 33.6% and 21.7%, respectively, whereas the Fe content was less than 0.2%.
